# The Prevalence of Multidrug-Resistant *Acinetobacter baumannii* and Its Vaccination Status among Healthcare Providers

**DOI:** 10.3390/vaccines11071171

**Published:** 2023-06-28

**Authors:** Ayman Elbehiry, Eman Marzouk, Ihab Moussa, Yazeed Mushayt, Ahmad Abdullah Algarni, Osama Ali Alrashed, Khalid Saad Alghamdi, Naif Ahmed Almutairi, Sulaiman Abdulaziz Anagreyyah, Anwar Alzahrani, Abdulaziz M. Almuzaini, Feras Alzaben, Meshal Abdullah Alotaibi, Suha Abdulaziz Anjiria, Akram Abu-Okail, Adil Abalkhail

**Affiliations:** 1 Department of Public Health, College of Public Health and Health Informatics, Qassim University, Al Bukayriyah 52741, Saudi Arabia; e.marzouk@qu.edu.sa (E.M.);; 2 Department of Bacteriology, Mycology and Immunology, Faculty of Veterinary Medicine, University of Sadat City, Sadat City 32511, Egypt; 3 Department of Botany and Microbiology, College of Science, King Saud University, Riyadh 11451, Saudi Arabia; 4 Department of Support Service, King Fahad Armed Hospital, Jeddah 23311, Saudi Arabia; 5 Family Medicine Department, King Fahad Armed Hospital, Jeddah 23311, Saudi Arabia; 6 Cardiac Center, King Fahad Armed Forces Hospital, Jeddah 23311, Saudi Arabia; 7 Department of Veterinary Medicine, College of Agriculture and Veterinary Medicine, Qassim University, Buraydah 52571, Saudi Arabia; 8 Department of Food Service, King Fahad Armed Hospital, Jeddah 23311, Saudi Arabia; 9 Academic Affairs, King Fahad Armed Hospital, Jeddah 23311, Saudi Arabia; 10 Pharmacy Department, King Abdullah Medical Complex, Jeddah 23816, Saudi Arabia

**Keywords:** multidrug-resistant *Acinetobacter baumannii*, prevalence, healthcare settings, vaccination strategies

## Abstract

There is growing concern among healthcare providers worldwide regarding the prevalence of multidrug-resistant *Acinetobacter baumannii* (*A. baumannii*). Some of the worst hospital-acquired infections, often in intensive care units (ICUs), are caused by this bacterial pathogen. In recent years, the rise in multidrug-resistant *A. baumannii* has been linked to the overuse of antimicrobial drugs and the lack of adequate infection control measures. Infections caused by this bacterial pathogen are the result of prolonged hospitalization and ICU stays, and they are associated with increased morbidity and mortality. This review outlines the epidemiology, risk factors, and antimicrobial resistance associated with *A. baumannii* in various countries, with a special focus on the Kingdom of Saudi Arabia. In response to the growing concern regarding this drug-resistant bacteria, fundamental information about its pathology has been incorporated into the development of vaccines. Although these vaccines have been successful in animal models, their effectiveness in humans remains unproven. The review will discuss the development of *A. baumannii* vaccines, potential related obstacles, and efforts to find an effective strategy against this pathogen.

## 1. Introduction

*Acinetobacter baumannii* is a widely distributed Gram-negative, non-fermentative, strictly non-motile, and oxidase-negative microbial pathogen that has been found in both natural and clinical environments [1,2]. It is also one of the six most significant pathogens in the ESKAPE group, which includes *Enterococcus faecium*, *Staphylococcus aureus*, *Klebsiella pneumoniae*, *A. baumannii*, *Pseudomonas aeruginosa*, and *Enterobacter* species [3,4]. According to the Infectious Disease Society of America, *A. baumannii* is a serious global nosocomial concern [3,5]. It is a primary cause of various infections, particularly in immunosuppressed individuals or those receiving mechanical ventilation in intensive care units (ICUs) [6]. The most common infections associated with *A. baumannii* include pneumonia, bacteremia, meningitis, respiratory tract infection, and urinary tract infection [5]. Over the past decade, mortality rates due to *A. baumannii* infections have increased in many regions worldwide, ranging from 30% to 75% [7,8,9]. Infections caused by *A. baumannii* are associated with various risk factors, including burns, preterm birth, prolonged hospital stays, mechanical ventilation, indwelling devices, and the extensive use of antibiotic therapy [10,11,12].

According to the World Health Organization, multidrug-resistant *A. baumannii* is one of the most critical pathogens due to its resistance to numerous antibiotic classes, particularly carbapenems and third-generation cephalosporins [7]. Several studies conducted in the Kingdom of Saudi Arabia have indicated the increasing resistance of *A. baumannii* isolates to various antibiotics [13]. During the coronavirus 2019 (COVID-19) pandemic, the healthcare crisis caused by *A. baumannii*, particularly the carbapenem-resistant strains, reached its peak in ICUs [14,15]. Therefore, developing novel vaccines and drug targets remains imperative to combat the relentless emergence of drug resistance in *A. baumannii* against currently available antibacterial drugs [16,17]. Developing novel vaccines and therapeutics is a time-consuming process that often spans several years [18,19]. Before advancing to clinical trials, vaccine candidates must undergo testing in experimental animals to assess reactogenicity and immune response [20]. Furthermore, it is essential to evaluate the pharmacological and toxicological properties of the targeted vaccine candidates to ensure their safety and efficacy [21]. This review investigates the spread of multidrug-resistant *A. baumannii* infections in healthcare settings in Saudi Arabia and explores potential measures to prevent their transmission.

## 2. *A. baumannii*’s Mechanisms of Antibiotic Resistance

Healthcare facilities are currently grappling with an outbreak of multidrug-resistant *A. baumannii* [13,22]. Surveillance investigations worldwide have reported a significant increase in antimicrobial resistance by *A. baumannii* across diverse regions, including South East Asia [23], the Arabian Peninsula [24], and other parts of the world [22]. This has posed substantial healthcare and financial challenges in Saudi Arabia, where numerous cases of nosocomial multidrug-resistant *A. baumannii* have been documented [13]. 

*A. baumannii*, like other Gram-negative bacteria, possesses various defense mechanisms that enable it to evade the bactericidal and bacteriostatic effects of antimicrobial agents. These mechanisms include efflux pumps, which expel antibiotics from the cell through efflux mechanisms, and modifications to outer-membrane proteins (OMPs) that reduce the permeability of porins [25]. Additionally, *A. baumannii* has been found to produce metallo-β-lactamase enzymes such as imipenemase (IMP), Verona integron-mediated metallo-β-lactamase (VIM), New Delhi metallo β-lactamase (NDM), and Seoul imipenemase (SIM). These enzymes can degrade antibiotics, rendering them ineffective [26]. However, the most crucial resistance mechanism employed by *A. baumannii* is the production of β-lactamases, particularly enzymes that hydrolyze carbapenems [14], penicillins [27], cephalosporins [27], and monobactams [14]. 

Studies conducted in Africa have revealed that the emergence of class D β-lactamases (OXA-23, -24, -51, and -58) and New Delhi metallo-β-lactamase (NDM-1) represents a significant mechanism of *A. baumannii* resistance. The presence of the *blaOXA-23*-like gene has been documented in several African countries, indicating its prevalence in these regions [28,29,30,31]. Furthermore, reports have highlighted the isolation of *A. baumannii* strains carrying Guiana extended-spectrum (GES) types, including *GES-11*, in Tunisia [32]. In addition to these mechanisms, other factors have contributed to multidrug resistance in this bacterium, such as the expression of efflux pumps (*TetA*, *TetB*, and *AdeABC*) [33] and the loss of or reduction in OMPs. In Saudi Arabia, El-Mahdy et al., (2017) conducted a study on carbapenem-resistant *A. calcoaceticus-baumannii* complex isolates from the eastern district. The findings revealed that these isolates carried the genes for carbapenem-resistant *blaOXA-23*-like and *ISAba1* phage, and nine of the isolates also possessed the blaADC gene [34]. 

## 3. The Epidemiology and Risk Factors Associated with *A. baumannii*

There have been numerous instances in which *A. baumannii* infections are transmitted from one medical facility to another due to the movement of infected patients. For instance, the Vietnam extended-spectrum-β-lactamase (VEB-1)-producing *A. baumannii* clone was responsible for spreading the *VEB-1* strain across 55 hospitals in northern and southeastern France. Furthermore, European clonal types I and II have been observed circulating in healthcare settings in Italy, with evidence suggesting the transfer of this strain from healthcare settings in the Mediterranean area to those in southwestern Germany [35,36,37]. Initial reports from hospitals in New York also associated outbreaks involving the *OXA-48* β-lactamase produced by *A. baumannii* with outbreaks detected within the hospitals. The movement of patients and healthcare staff between healthcare providers has facilitated the spread of multidrug-resistant *A. baumannii* [38].

In Saudi Arabia, several investigations have been conducted to identify potential risk factors for *A. baumannii* infections [13,39,40,41,42]. Several studies have indicated that *A. baumannii* endemic strains may spread to tracheal secretions in patients undergoing mechanical ventilation [43]. Patients aged 60 or older and those receiving prolonged oxygen therapy are at a higher risk of developing ventilator-associated pneumonia caused by *A. baumanni* [41,44]. The colonization of the intestinal tract by *A. baumannii* in hospitalized patients is a potential risk factor for antibiotic resistance and outbreaks of serious infections [45]. Additionally, patients with chronic conditions that compromise their immune systems, such as diabetes, malignancy, kidney disease, and persistent lung disease, are particularly susceptible [39,46,47]. An earlier study showed that out of 72 patients infected with *A. baumannii*, 36% had underlying illnesses, and 11% were diabetics [48]. 

## 4. The Impact of *Acinetobacter baumannii* among Saudi Healthcare Facilities

**Figure 1:**
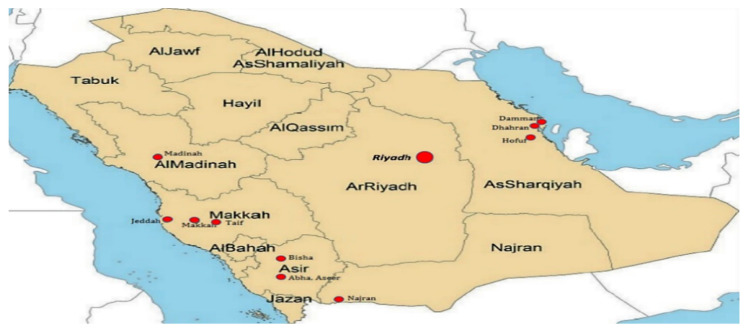
The Saudi Arabian map showing administrative regions and hospitals (highlighted in red).

Saudi Arabia is geographically divided into 13 administrative regions (Figure 1), each with its own political authority. With a combined population of over 36 million as of 2023, the country accommodates millions of Muslims from around the globe during the annual Hajj pilgrimage in the cities of Makkah and Madinah. This massive congregation poses a significant risk for the transmission of infectious diseases. Consequently, Saudi Arabia serves as a central hub for the international dissemination of multidrug-resistant (MDR) strains [49,50,51].

According to the Saudi Ministry of Health, Acinetobacter infections are among the leading causes of healthcare expenditure [52]. The negative impact on outcomes and high mortality rates associated with Acinetobacter infections are due to inappropriate therapies and limited therapeutic options, especially in ICU patients [53,54]. Saudi Arabia has observed an increase in carbapenem-resistant *A. baumannii* over the past decade compared to the 1990s [24]. A previous study reported that between 2005 and 2009, 26.5% of ventila-tor-associated pneumonia cases in Riyadh, Saudi Arabia, were attributed to Acinetobacter species [52]. In another investigation [55], the incidence of multidrug-resistant *A. baumannii* was 92.1%, the highest percentage recorded to date. A study published in 2015 unveiled the high resistance of *A. baumannii* infections in the El-Qassim region of Saudi Arabia to multiple antibiotics [56]. Similarly, an investigation conducted in Jeddah found that multidrug-resistant *A. baumannii* was present in 55% of patients in 2010 and 67% of patients in 2013 [42]. Table 1 provides a comprehensive overview of the incidence of multidrug-resistant *A. baumannii* over the past 15 years, with each investigation indicating a progressively higher prevalence. According to Table 1, *A. baumannii* displayed a variable degree of resistance, up to 100%, against carbapenems, penicillins, and cephalosporins during surveys conducted between 2004 and 2015 throughout Saudi Arabia.

## 5. Controlling Multidrug-Resistant *Acinetobacter baumannii* Outbreaks in Healthcare Settings

There have been numerous instances worldwide where multidrug-resistant *A. baumannii* has been successfully controlled through the implementation of various methods [73]. The concept of a source control program encompasses antibiotic stewardship, hand hygiene, adherence to contact precautions, education, thorough environmental cleaning, and vaccination strategies [74]. To effectively manage multidrug-resistant *A. baumannii*, it is imperative to gather microbial susceptibility profile data from specific regions or hospitals and establish rapid and cost-effective testing methods [75]. Several measures need to be taken to address this issue, including providing healthcare professionals with training on conducting laboratory tests and enhancing research skills among caregivers, particularly those working in the ICU [76,77].

Despite colistin being one of the few remaining effective treatments for multidrug-resistant *A. baumannii*, its efficacy has been compromised due to the degradation of lipopolysaccharide (LPS) and the emergence of lipid A target modifications [78]. Consequently, there is an urgent need for novel therapeutic approaches to tackle these challenges. Different organizations have recommended various strategies as the primary treatment for multidrug-resistant *A. baumannii* infections [79,80,81]. In line with Shields et al.’s recommendation, a combination of high-dose ampicillin–sulbactam with cefiderocol, polymyxin B, or tigecycline is recommended as the prescribed regimen in 2023 [82].

Alternative therapies, such as phage therapy, are increasingly being used to treat multidrug-resistant *A. baumannii* infections [83,84]. While phage therapy offers several advantages, certain aspects still require further clarification. These include the long-term viability of formulation and manufacturing scale, the potential resurgence of bacteriophage resistance, and the overall impact on gut microbiota [85]. In the future, virulent phages may emerge as potent bactericidal agents, potentially replacing antibiotics; however, the rapid emergence of phage resistance significantly impedes their widespread and continuous utilization [86].

## 6. Vaccine Development against *Acinetobacter baumannii*

*A. baumannii* vaccine is currently unavailable despite several vaccine candidates being tested [87]. Vaccines targeting *A. baumannii* are crucial for safeguarding individuals with weakened immune systems and patients with underlying medical conditions [88]. In the aftermath of McConnell et al.’s 2013 report [89], many laboratories are working on vaccines targeted to specific populations. Given the similarity among clinical isolates of *A. baumannii*, vaccines targeting conserved antigens may offer effective protection [90]. In the past decade, several multivalent vaccines have been considered, including live-attenuated strains, ghost bacteria, outer-membrane vesicles, and DNA (Table 2). Extensive studies have been conducted on the types and characteristics of potential vaccine candidates for *A. baumannii*. In this section of the review article, our objective is to assess each of these platforms, describe the experiments conducted with rodents to evaluate their ability to elicit immune responses, and assess their protective efficacy.

### 6.1. Live-Attenuated Vaccines

Live-attenuated vaccines are produced by modifying bacteria to reduce their harmfulness, allowing the immune system to combat the infectious agent without causing illness [90]. Since live-attenuated strains display antigens similar to those found in fully pathogenic strains, they provide multiple targets for both immunoglobulin and cellular immune responses [97]. However, a drawback of live-attenuated vaccines is their potential to regain their harmful properties through horizontal gene transmission or spontaneous mutation [98]. Furthermore, it remains unclear whether live-attenuated vaccinations can be administered to individuals with weakened immune systems, who are most susceptible to *A. baumannii* infection. Recently, a live-attenuated vaccine against *A. baumannii* was developed based on a D-glutamate strain [98]. By modifying the *murI1* and *murI2* genes, a significantly less virulent strain of *A. baumannii* was created, which still elicited both antibody- and cell-mediated immune responses and improved survival in immunized animals exposed to multiple pathogenic strains of *A. baumannii* [98]. Additionally, the immunity induced by an *A. baumannii* strain lacking thioredoxin (∆trxA) was investigated [99]. Thioredoxins are vital in regulating redox balance and oxidative stress in bacteria. It was observed that a clinical isolate of *A. baumannii* lacking thioredoxin did not cause illness in mice vaccinated with the ∆trxA strain [99,100].

### 6.2. Outer-Membrane Vesicles and Complex-Based Vaccines

Virulent Gram-negative bacteria release outer-membrane vesicles (OMVs), which are non-infectious particles with diameters ranging from 10 to 300 nanometers [90,101]. *A. baumannii* is one of the bacteria that produce OMVs [102]. These spherical structures are typically composed of phospholipids, protein molecules, DNA, and LPS [103]. Alaniz et al. [104] suggest that OMVs are involved in delivering virulence factors to host cells, thereby initiating the infection process. McBroom and Kuehn [105] found that OMV production increases when stress levels are high and severe environments are present, such as during an infection. While the exact mechanism of OMV formation is yet to be determined, Deatherage and colleagues suggested that OMVs are generated when the density of outer-membrane–peptidoglycan connections is temporarily reduced. Furthermore, the researchers discovered that OMPs in OMVs contain specific domains that can interact with peptidoglycan and influence their development [106]. The effectiveness of OMV-based vaccines has been demonstrated in various studies conducted in animal models, with a variety of microorganisms, including *Escherichia coli* [107,108], *Bordetella pertussis* [109], and *Burkholderia pseudomallei* [110,111].

When considering a vaccination based on OMVs, it is important to remember that LPS plays a significant role in immunogenicity, as well as the potential side effects that result from the excessive presence of endotoxin. Significant progress has been made in addressing some of these issues by isolating OMVs from the *A. baumannii* strain IB010 [93]. It has been reported that this strain cannot produce LPS due to a mutation in the *lpxD* gene [93]. A study investigating the protective effects of LPS-*A. baumannii* outer-membrane complexes (OMCs) utilized the same strain. Mice vaccinated with the LPS-deficient OMCs showed little to no toxicity, similar to that of the LPS-deficient OMVs. On day 7 of the same experiment, mice vaccinated with LPS-deficient OMCs had a 60% survival rate, while mice vaccinated with LPS-deficient OMCs supplemented with exogenous LPS had a 95% survival rate [112]. A noticeable difference was observed between the two groups compared to the untreated control group, in which no survivors were found [112]. These investigations suggest that the LPS antigen contained within an OMV-based vaccination plays a crucial role in protecting against infection, despite the potential absence of LPS, which could help prevent excessive inflammation levels.

### 6.3. Nucleic-Acid-Based Vaccines

The concept of nucleic acid vaccines has gained considerable attention as a potential platform for vaccinations in recent years. However, very few studies have been conducted on nucleic acid vaccines intended to protect against *A. baumannii* [113]. Recent studies have demonstrated that DNA vaccines carrying the *OmpA* gene from *A. baumannii* can protect mice in animal pneumonia models from potentially fatal bacterial infections [114,115]. Researchers have found that immunization with a plasmid-vector-mediated vaccine (*pVAX1*) carrying the *OmpA* and proteoglycan-associated lipoprotein (*PAL*) genes of *A. baumannii* is effective against a lethal pulmonary challenge with four heterologous strains, resulting in 50% survival rates. Additionally, a decrease in inflammatory cytokines and the infiltration of inflammatory cells were observed in the bronchoalveolar lavage (BAL) [114], along with a strong humoral response, mixed cellular responses from T-helper cells (Th1/Th2/Th17), and a reduction in microbial burdens. Another vaccine utilizing the alternative plasmid-encoded *OmpA* expressed through the eukaryotic expression vector pBudCE4.1 protected 16% of mice from a lung infection for up to 15 days following a challenge. This vaccine also showed modest responses in Interleukin (IL-2, IL-4, and IL-12) and interferons (IFNs) [116]. These DNA vaccination strategies are still in their early stages but show promise as potentially safe and affordable approaches for administering multivalent vaccines.

### 6.4. Bacterial-Ghost-Based Vaccines

Bacterial ghosts (BGs) are initially living bacteria that undergo a process in which all their cytosolic components are removed, leaving only their outer membranes intact. Due to their non-living nature, BGs possess several inherent advantages, including the inability to revert to a pathogenic phenotype when utilized [90]. BGs can also serve as self-adjuvants and can be engineered to contain specific proteins, DNA, medications, or other small molecules to enhance their efficacy. However, while BGs maintain their original outer membrane, an excessive amount of natural LPS may lead to increased inflammation [97]. In an experiment, Sprague Dawley rats were vaccinated with *A. baumannii* BGs (strain Ali190) via oral, subcutaneous, intramuscular, or intraperitoneal administration before being infected with 10^8^ CFU of the homologous *A. baumannii* strain [96]. *A. baumannii* BGs proved highly effective in protecting immunized rats against *A. baumannii* infection in all administration modes, except for oral vaccination, which resulted in a 67% survival rate [96]. However, despite the favorable outcomes of this initial study, it is crucial to investigate whether *A. baumannii* BGs can confer immunity against a wide range of *A. baumannii* strains.

## 7. Lessons Learned and Future Perspectives

Despite efforts made, the development of vaccines against *A. baumannii* has fallen behind that of nosocomial pathogens such as *Clostridium difficile*, *Pseudomonas aeruginosa*, and *Staphylococcus aureus* [95]. No vaccine against *A. baumannii* has undergone clinical trials, indicating the challenges associated with creating a safe and effective vaccine against this pathogen.

### 7.1. Technology of Modern Vaccines

Currently, research on anti-*A. baumannii* vaccine is still in its early stages. Most studies have focused on traditional methods and platforms, such as subunit and killed vaccines. However, there are ongoing explorations of novel antigen transporters and enhanced delivery mechanisms, including nucleic acid vaccines, virus vectors, conjugated carriers, and co-delivery [21,113,117]. It is worth noting that mRNA and DNA vaccine technologies have gained significant recognition due to the competition surrounding COVID-19 vaccines. Therefore, we can anticipate the increasing importance of nucleic-acid-based vaccines in treating *A. baumannii* and other infections in the coming years [113]. Additionally, proteomic approaches and reverse vaccination offer the benefit of utilizing bioinformatics to systematically evaluate and select potential immunogens for vaccine candidates in silico [113]. This method may be employed in the future to identify antigens and design vaccines that protect against *A. baumannii* infections.

### 7.2. The Long-Term Effects of Cellular Immunity

A promising vaccine candidate against *A. baumannii* should demonstrate its protective effects in preclinical trials by improving the survival rates of immunized animals compared to non-immunized ones, reducing bacterial burdens within organs, and suppressing the levels of inflammatory markers in the serum [113]. While vaccination and infection have been extensively researched, less attention has been given to studying cell-mediated immunity following vaccination. To formulate effective vaccines against *A. baumannii*, researchers need to align their assessment procedures, including animal models, intervals between vaccinations and challenges, challenge strains, doses, and administration methods. This harmonization will enable more accurate comparisons of experimental vaccines developed in different laboratories [95].

### 7.3. Cross-Protective Multivalent Vaccines

Vaccine candidates have been evaluated not only against homologous strains but also against challenges posed by other clinical isolates to demonstrate a broad level of protection. However, the degree of risk reduction varied significantly depending on the specific strain, vaccination protocol, and/or challenge dose [113]. Consequently, it is necessary to further assess the protective properties of these vaccines, especially when dealing with highly infectious strains at extremely high doses. Developing a vaccine that offers broad or diverse protection is challenging due to strain-dependent variation in selected antigens such as *OmpA* [118,119]. The complete ATCC 179878 genome revealed the presence of 28 putative alien islands, suggesting the potential for repeated infections and the difficulty of developing a vaccine [120]. *A. baumannii* has a high propensity for acquiring foreign DNA, leading to the emergence of novel genetic variants. Therefore, it is crucial to select conserved antigens and design vaccines with a wide range of components to provide adequate protection.

## 8. Limitations

This review has limitations, such as focusing solely on the prevalence of multidrug-resistant *A. baumannii* in Saudi Arabia without addressing other countries. To provide a more comprehensive view, further studies should be conducted in other countries to compare the data. This will enable us to better understand the global distribution of multidrug-resistant *A. baumannii* and the strategies needed to control its spread. Additionally, we did not highlight any of the virulence factors associated with *A. baumannii* infection, which should be addressed. Identifying these virulence factors is essential for understanding how this bacterium causes infections and developing strategies for their prevention and treatment. Moreover, this review did not discuss the potential risks and safety issues associated with developing a vaccine against multidrug-resistant *A. baumannii*, nor did it provide any information regarding the costs or potential barriers associated with its development and implementation.

## 9. Conclusions

The review shows that multidrug-resistant *A. baumannii* infections are prevalent in Saudi Arabia and that combination therapies are a key strategy for overcoming drug resistance. In particular, antibiotic stewardship is essential for preventing the emergence of drug-resistant strains, and multivalent vaccines composed of outer-membrane vesicles (OMVs), bacterial ghosts (BGs), or multiple subunit vaccines hold promise in reducing these infections. The development of vaccines has undergone significant changes due to advancements in technology, particularly in the wake of COVID-19 vaccine development.

## Figures and Tables

**Table 1:** An evaluation of the resistance rates of *A. baumannii* to β-lactam antibiotics was carried out in hospitals across Saudi Arabia (the numbers in the table represent the percentage of *A. baumannii* resistant to antibiotics).

Area	Region	Survey Year	β-Lactam Antibiotics									Reference
			Carbapenems			Penicillins			Cephalosporins			
			Aztreonam	Imipenem	Meropenem	Amoxicillin-clavulanic acid	Piperacillin	Piperacillin-tazobactam	Cefoxitin	Cefepime	Ceftazidime	
Central	Riyadh	2006	0	36	19	-	-	-	-	-	75	[40]
		2006–2008	-	79.1	92.1	-	-	-	-	99	-	[55]
		2009	83	89	91	96	-	93	-	-	88	[57]
		2010	78	89	89	-	-	-	78	78	-	[58]
		2011	100	64	-	-	-	-	100	100	-	[59]
		2006–2014	-	77	88.5	-	-	-	-	97	96.2	[60]
		2013–2015	-	73	82	-	-	-	-	68	92	[61]
	Qassim	2011	-	9	-	-	-	42	-	67	89	[62]
		2011–2015	-	-	-	100	100	-	-	-	100	[56]
West	Makkah	2004–2005	90	14	-	87	73	8	92	45	79	[63]
		2005–2006	95.3	46	28	93	-	-	98	-	87	[64]
		2015	-	90	64	-	-	-	-	77	77	[65]
	Madinah	2014	96	-	-	93.7	71	-	100	-	100	[66]
	Jeddah	1999–2000	81	15	-	71	60	-	-	-	59	[67]
		2012	-	66	-	-	-	-	-	27	25	[13]
East	Dammam	2010–2012	-	33	33.3	-	-	33.3	-	74	85.1	[68]
		2014	-	-	-	-	-	-	100	-	-	[45]
		2014	-	100	100	-	-	-	-	100	100	[34]
	Khobar	2015	-	-	-	-	-	-	-	68	71	[69]
South	Aseer	2011–2012	-	3	-	-	-	-	-	-	39	[70]
		2013–2014	0	52	50	-	81.5	-	-	90	-	[71]
		2014–2015	92	95.5	100	-	97.1	-	-	97	93	[41]
	Najran	2012–2013	-	7.4	1.5	-	-	32.4	-	46	91	[72]

**Table 2:** Types and characteristics of potential vaccine candidates for *Acinetobacter baumannii*.

Vaccine Type	Formula for a Vaccine	Examples	Reference
Live-attenuated	Formalin-inactivated cells with aluminum-based adjuvant and alhydrogel^®^ adjuvant 2%	Inactivated lipopolysaccharide-deficient whole cells	[91]
		Inactivated *A. baumannii Lac4 strain*	[92]
Outer-membrane vesicles (OMVs) and complex (OMCs)	OMVs in phosphate buffer saline with an aluminum phosphate adjuvant	OMVs	[93]
	OMCs in phosphate buffer saline with an aluminum phosphate adjuvant	OMCs	[93]
		LPS-free OMVs	[93]
DNA	3 µg of rickettsial outer-membrane protein A (rOmpA) in 0.1% aluminum hydroxide	*OmpA*	[94]
		*OmpA* and *PAL*	[95]
Bacterial ghosts	*A. baumannii* Ali190 being incubated in a solution of hydrogen peroxide, sodium hydroxide, and sodium carbonate	LPS/surface	[96]

## Data Availability

Not applicable.

## References

[1] Nguyen M., Joshi S. (2021). Carbapenem resistance in *Acinetobacter baumannii*, and their importance in hospital-acquired infections: A scientific review. J. Appl. Microbiol..

[2] Carvalheira A., Silva J., Teixeira P. (2021). Acinetobacter spp. in food and drinking water–A review. Food Microbiol..

[3] Katic V., Todorovic J., Nagorni A., Nagorni I., Micev M. (2017). A Case of Kaposi’s Sarcoma of the Rectum in a Homosexual Male with HIV–AIDS. Infect. Dis. Ther..

[4] Bhatia P., Sharma A., George A.J., Anvitha D., Kumar P., Dwivedi V.P., Chandra N.S. (2021). Antibacterial activity of medicinal plants against ESKAPE: An update. Heliyon.

[5] Boucher H.W., Talbot G.H., Bradley J.S., Edwards J.E., Gilbert D., Rice L.B., Scheld M., Spellberg B., Bartlett J. (2009). Bad bugs, no drugs: No ESKAPE! An update from the Infectious Diseases Society of America. Clin. Infect. Dis..

[6] Biderman P., Bugaevsky Y., Ben-Zvi H., Bishara J., Goldberg E. (2015). Multidrug-resistant *Acinetobacter baumannii* infections in lung transplant patients in the cardiothoracic intensive care unit. Clin. Transplant..

[7] Agyepong N., Fordjour F., Owusu-Ofori A. (2023). Multidrug Resistant *Acinetobacter baumannii* in Healthcare Settings in Africa. Front. Trop. Dis..

[8] Brotfain E., Borer A., Koyfman L., Saidel-Odes L., Frenkel A., Gruenbaum S.E., Rosenzweig V., Zlotnik A., Klein M. (2017). Multidrug resistance Acinetobacter bacteremia secondary to ventilator-associated pneumonia: Risk factors and outcome. J. Intensive Care Med..

[9] Garnacho-Montero J., Gutiérrez-Pizarraya A., Díaz-Martín A., Cisneros-Herreros J.M., Cano M.E., Gato E., de Alegría C.R., Fernández-Cuenca F., Vila J., Martínez-Martínez L. (2016). *Acinetobacter baumannii* in critically ill patients: Molecular epidemiology, clinical features and predictors of mortality. Enferm. Infecc. Y Microbiol. Clin..

[10] Antunes L.C., Visca P., Towner K.J. (2014). *Acinetobacter baumannii*: Evolution of a global pathogen. Pathog. Dis..

[11] Visca P., Seifert H., Towner K.J. (2011). Acinetobacter infection–an emerging threat to human health. IUBMB Life.

[12] Elbehiry A., Marzouk E., Moussa I.M., Dawoud T.M., Mubarak A.S., Al-Sarar D., Alsubki R.A., Alhaji J.H., Hamada M., Abalkhail A. (2021). *Acinetobacter baumannii* as a community foodborne pathogen: Peptide mass fingerprinting analysis, genotypic of biofilm formation and phenotypic pattern of antimicrobial resistance. Saudi J. Biol. Sci..

[13] Ibrahim M.E. (2019). Prevalence of *Acinetobacter baumannii* in Saudi Arabia: Risk factors, antimicrobial resistance patterns and mechanisms of carbapenem resistance. Ann. Clin. Microbiol. Antimicrob..

[14] Kyriakidis I., Vasileiou E., Pana Z.D., Tragiannidis A. (2021). *Acinetobacter baumannii* antibiotic resistance mechanisms. Pathogens.

[15] Thoma R., Seneghini M., Seiffert S.N., Vuichard Gysin D., Scanferla G., Haller S., Flury D., Boggian K., Kleger G.-R., Filipovic M. (2022). The challenge of preventing and containing outbreaks of multidrug-resistant organisms and Candida auris during the coronavirus disease 2019 pandemic: Report of a carbapenem-resistant *Acinetobacter baumannii* outbreak and a systematic review of the literature. Antimicrob. Resist. Infect. Control.

[16] Suranadi I.W., Fatmawati N., Aryabiantara I.W., Sinardja C.D., Saputra D.J., Senapathi T., Widnyana I., Nada I., Astuti M., Kumara V. (2019). *Acinetobacter baumannii* is an opportunistic pathogen as an MDRO in ICU. Bali. J. Anesth..

[17] Zhang T., Xu X., Xu C.-F., Bilya S.R., Xu W. (2021). Mechanical ventilation-associated pneumonia caused by *Acinetobacter baumannii* in Northeast China region: Analysis of genotype and drug resistance of bacteria and patients’ clinical features over 7 years. Antimicrob. Resist. Infect. Control.

[18] Kim Y.C., Dema B., Reyes-Sandoval A. (2020). COVID-19 vaccines: Breaking record times to first-in-human Trials. npj Vaccines.

[19] Matthews H., Hanison J., Nirmalan N. (2016). “Omics”-informed drug and biomarker discovery: Opportunities, challenges and future perspectives. Proteomes.

[20] Leroux-Roels G., Bonanni P., Tantawichien T., Zepp F. (2011). Vaccine development. Perspect. Vaccinol..

[21] Tan Y.C., Lahiri C. (2022). Promising *Acinetobacter baumannii* vaccine candidates and drug targets in recent years. Front. Immunol..

[22] Bertrand X., Dowzicky M.J. (2012). Antimicrobial susceptibility among gram-negative isolates collected from intensive care units in North America, Europe, the Asia-Pacific Rim, Latin America, the Middle East, and Africa between 2004 and 2009 as part of the Tigecycline Evaluation and Surveillance Trial. Clin. Ther..

[23] Howard A., O’Donoghue M., Feeney A., Sleator R.D. (2012). *Acinetobacter baumannii*: An emerging opportunistic pathogen. Virulence.

[24] Zowawi H.M., Balkhy H.H., Walsh T.R., Paterson D.L. (2013). β-Lactamase production in key gram-negative pathogen isolates from the Arabian Peninsula. Clin. Microbiol. Rev..

[25] Wilson D.N. (2014). Ribosome-targeting antibiotics and mechanisms of bacterial resistance. Nat. Rev. Microbiol..

[26] Cornaglia G., Giamarellou H., Rossolini G.M. (2011). Metallo-β-lactamases: A last frontier for β-lactams?. Lancet Infect. Dis..

[27] Dijkshoorn L., Nemec A., Seifert H. (2007). An increasing threat in hospitals: Multidrug-resistant *Acinetobacter baumannii*. Nat. Rev. Microbiol..

[28] Olaitan A.O., Berrazeg M., Fagade O.E., Adelowo O.O., Alli J.A., Rolain J.M. (2013). Emergence of multidrug-resistant *Acinetobacter baumannii* producing OXA-23 carbapenemase, Nigeria. Int. J. Infect. Dis..

[29] Bakour S., Olaitan A.O., Ammari H., Touati A., Saoudi S., Saoudi K., Rolain J.-M. (2015). Emergence of colistin-and carbapenem-resistant *Acinetobacter baumannii* ST2 clinical isolate in Algeria: First case report. Microb. Drug Resist..

[30] Mugnier P.D., Poirel L., Naas T., Nordmann P. (2010). Worldwide dissemination of the blaOXA-23 Carbapenemase gene of *Acinetobacter baumannii*. Emerg. Infect. Dis..

[31] Nogbou N.-D., Phofa D.T., Nchabeleng M., Musyoki A.M. (2021). Investigating multi-drug resistant Acinetobacter baumannii isolates at a tertiary hospital in Pretoria, South Africa. Indian J. Med. Microbiol..

[32] Chihi H., Bonnin R., Bourouis A., Mahrouki S., Besbes S., Moussa M.B., Belhadj O., Naas T. (2016). GES-11-producing *Acinetobacter baumannii* clinical isolates from Tunisian hospitals: Long-term dissemination of GES-type carbapenemases in North Africa. J. Glob. Antimicrob. Resist..

[33] Pannek S., Higgins P.G., Steinke P., Jonas D., Akova M., Bohnert J.A., Seifert H., Kern W.V. (2006). Multidrug efflux inhibition in *Acinetobacter baumannii*: Comparison between 1-(1-naphthylmethyl)-piperazine and phenyl-arginine-β-naphthylamide. J. Antimicrob. Chemother..

[34] El-Mahdy T.S., Al-Agamy M.H., Al-Qahtani A.A., Shibl A.M. (2017). Detection of bla OXA-23-like and bla NDM-1 in *Acinetobacter baumannii* from the Eastern Region, Saudi Arabia. Microb. Drug Resist..

[35] Higgins P.G., Dammhayn C., Hackel M., Seifert H. (2010). Global spread of carbapenem-resistant *Acinetobacter baumannii*. J. Antimicrob. Chemother..

[36] D’arezzo S., Capone A., Petrosillo N., Visca P. (2009). Epidemic multidrug-resistant *Acinetobacter baumannii* related to European clonal types I and II in Rome (Italy). Clin. Microbiol. Infect..

[37] Naas T., Coignard B., Carbonne A., Blanckaert K., Bajolet O., Bernet C., Verdeil X., Astagneau P., Desenclos J., Nordmann P. (2006). French Nosocomial Infection Early Warning Investigation and Surveillance Network. VEB-1 Extended-spectrum beta-lactamase-producing *Acinetobacter baumannii*, France. Emerg. Infect. Dis..

[38] Alsan M., Klompas M. (2010). *Acinetobacter baumannii*: An emerging and important pathogen. J. Clin. Outcomes Manag. JCOM.

[39] Al-Anazi K.A., Al-Jasser A.M. (2014). Infections caused by *Acinetobacter baumannii* in recipients of hematopoietic stem cell transplantation. Front. Oncol..

[40] Al-Obeid S., Jabri L., Al-Agamy M., Al-Omari A., Shibl A. (2015). Epidemiology of extensive drug resistant *Acinetobacter baumannii* (XDRAB) at Security Forces Hospital (SFH) in Kingdom of Saudi Arabia (KSA). J. Chemother..

[41] Al Bshabshe A., Joseph M.R., Al Hussein A., Haimour W., Hamid M.E. (2016). Multidrug resistance Acinetobacter species at the intensive care unit, Aseer Central Hospital, Saudi Arabia: A one year analysis. Asian Pac. J. Trop. Med..

[42] Al Mobarak M.F., Matbuli R.M., Meir H., Al Gehani N., El Toukhy A.A.M., Al Qureshey K.F., Mutwalli A.H., Abdulaziz A.M., Hadhoud A. (2014). Antimicrobial Resistance Patterns among *Acinetobacter baumannii* Isolated from King Abdulaziz Hospital, Jeddah, Saudi Arabia: Four-Year Surveillance Study (2010–2013). Egypt. J. Med. Microbiol..

[43] Mah M.W., Memish Z.A., Cunningham G., Bannatyne R.M. (2001). Outbreak of *Acinetobacter baumannii* in an intensive care unit associated with tracheostomy. Am. J. Infect. Control.

[44] El-Saed A., Balkhy H.H., Al-Dorzi H.M., Khan R., Rishu A.H., Arabi Y.M. (2013). Acinetobacter is the most common pathogen associated with late-onset and recurrent ventilator-associated pneumonia in an adult intensive care unit in Saudi Arabia. Int. J. Infect. Dis..

[45] Aljindan R., Bukharie H., Alomar A., Abdalhamid B. (2015). Prevalence of digestive tract colonization of carbapenem-resistant *Acinetobacter baumannii* in hospitals in Saudi Arabia. J. Med. Microbiol..

[46] Al-Gethamy M.M., Faidah H.S., Adetunji H.A., Haseeb A., Ashgar S.S., Mohanned T.K., Mohammed A.-H., Khurram M., Hassali M.A. (2017). Risk factors associated with multi-drug-resistant *Acinetobacter baumannii* nosocomial infections at a tertiary care hospital in Makkah, Saudi Arabia-a matched case–control study. J. Int. Med. Res..

[47] Nurain A.M., Bilal N.E., Ibrahim M.E. (2015). The frequency and antimicrobial resistance patterns of nosocomial pathogens recovered from cancer patients and hospital environments. Asian Pac. J. Trop. Biomed..

[48] Khan M.A., Mahomed M.F., Ashshi A.M., Faiz A. (2012). Drug resistance patterns of *Acinetobacter baumannii* in Makkah, Saudi Arabia. Pak. J. Med. Res..

[49] Abbott I., Cerqueira G.M., Bhuiyan S., Peleg A.Y. (2013). Carbapenem resistance in *Acinetobacter baumannii*: Laboratory challenges, mechanistic insights and therapeutic strategies. Expert Rev. Anti-Infect. Ther..

[50] Abdalhamid B., Hassan H., Itbaileh A., Shorman M. (2014). Characterization of carbapenem-resistant *Acinetobacter baumannii* clinical isolates in a tertiary care hospital in Saudi Arabia. New Microbiol..

[51] Raees F., Harun A., Ahmed A., Deris Z.Z. (2022). Antibiotic resistance, genotype and clinical significance of *Acinetobacter baumannii* in Saudi Arabia. Bangladesh J. Med. Sci..

[52] Kharaba A., Hussein M.A.A., Al-Hameed F.M., Mandourah Y., Almekhlafi G.A., Algethamy H., Hamdan A., Azem M.A., Fatani J., al Beshabshe A. (2019). *Acinetobacter baumannii* in Saudi Arabia: The new growing threat. Saudi Crit. Care J..

[53] Bassetti M., Righi E., Vena A., Graziano E., Russo A., Peghin M. (2018). Risk stratification and treatment of ICU-acquired pneumonia caused by multidrug-resistant/extensively drug-resistant/pandrug-resistant bacteria. Curr. Opin. Crit. Care.

[54] Russo A., Giuliano S., Ceccarelli G., Alessandri F., Giordano A., Brunetti G., Venditti M. (2018). Comparison of septic shock due to multidrug-resistant *Acinetobacter baumannii* or *Klebsiella pneumoniae* carbapenemase-producing *K. pneumoniae* in intensive care unit patients. Antimicrob. Agents Chemother..

[55] Aly M., Tayeb H., Al Johani S., Alyamani E., Aldughaishem F., Alabdulkarim I., Balkhy H. (2014). Genetic diversity of OXA-51-like genes among multidrug-resistant *Acinetobacter baumannii* in Riyadh, Saudi Arabia. Eur. J. Clin. Microbiol. Infect. Dis..

[56] Abdallah E.M., Ahamed F., Al-Omari A.S. (2015). Antibiotic susceptibility patterns of some clinical isolates from al-Rass general hospital. Int. J. Biosci..

[57] Saeed N.K., Kambal A.M., El-Khizzi N.A. (2010). Antimicrobial-resistant bacteria in a general intensive care unit in Saudi Arabia. Saudi Med. J..

[58] Al-Agamy M.H., Jeannot K., El-Mahdy T.S., Shibl A.M., Kattan W., Plésiat P., Courvalin P. (2017). First detection of GES-5 carbapenemase-producing *Acinetobacter baumannii* isolate. Microb. Drug Resist..

[59] Al-Agamy M.H., Shibl A.M., Ali M.S., Khubnani H., Radwan H.H., Livermore D.M. (2014). Distribution of β-lactamases in carbapenem-non-susceptible *Acinetobacter baumannii* in Riyadh, Saudi Arabia. J. Glob. Antimicrob. Resist..

[60] Aly M., Alsoud N.A., Elrobh M., Al Johani S., Balkhy H. (2016). High prevalence of the PER-1 gene among carbapenem-resistant *Acinetobacter baumannii* in Riyadh, Saudi Arabia. Eur. J. Clin. Microbiol. Infect. Dis..

[61] Al-Otaibi F.E., Bukhari E.E., Badr M., Alrabiaa A.A. (2016). Prevalence and risk factors of Gram-negative bacilli causing blood stream infection in patients with malignancy. Saudi Med. J..

[62] Said K.B., Al-Jarbou A.N., Alrouji M., Al-Harbi H.O. (2014). Surveillance of antimicrobial resistance among clinical isolates recovered from a tertiary care hospital in Al Qassim, Saudi Arabia. Int. J. Health Sci..

[63] Asghar A.H., Ashshi A.M., Azhar E.I., Bukhari S.Z., Zafar T.A., Momenah A.M. (2011). Profile of bacterial pneumonia during Hajj. Indian J. Med. Res..

[64] Asghar A.H., Faidah H.S. (2009). Frequency and antimicrobial susceptibility of gram-negative bacteria isolated from 2 hospitals in Makkah, Saudi Arabia. Saudi Med. J..

[65] Haseeb A., Faidah H.S., Bakhsh A.R., Al Malki W.H., Elrggal M.E., Saleem F., ur Rahman S., Khan T.M., Hassali M.A. (2016). Antimicrobial resistance among pilgrims: A retrospective study from two hospitals in Makkah, Saudi Arabia. Int. J. Infect. Dis..

[66] El-Ageery S., Al-Hazmi S. (2014). Microbiological and molecular detection of VIM-1 metallo beta lactamase-producing *Acinetobacter baumannii*. Eur. Rev. Med. Pharmacol. Sci..

[67] Eltahawy A., Khalaf R. (2001). Antibiotic resistance among gram-negative non-fermentative bacteria at a teaching hospital in Saudi Arabia. J. Chemother..

[68] Dhabaan G.N., AbuBakar S., Cerqueira G.M., Al-Haroni M., Pang S.P., Hassan H. (2016). Imipenem treatment induces expression of important genes and phenotypes in a resistant *Acinetobacter baumannii* isolate. Antimicrob. Agents Chemother..

[69] Abdalhamid B., Elhadi N., Alabdulqader N., Alsamman K., Aljindan R. (2016). Rates of gastrointestinal tract colonization of carbapenem-resistant Enterobacteriaceae and *Pseudomonas aeruginosa* in hospitals in Saudi Arabia. New Microb. New Infect..

[70] Abdalla N.M., Osman A.A., Haimour W.O., Sarhan M., Mohammed M.N., Zyad E.M., Al-Ghtani A.M. (2013). Antimicrobial susceptibility pattern in nosocomial infections caused by Acinetobacter species in Asir Region, Saudi Arabia. Pak. J. Biol. Sci. PJBS.

[71] Elabd F.M., Al-Ayed M.S., Asaad A.M., Alsareii S.A., Qureshi M.A., Musa H.A.-A. (2015). Molecular characterization of oxacillinases among carbapenem-resistant *Acinetobacter baumannii* nosocomial isolates in a Saudi hospital. J. Infect. Public Health.

[72] Asaad A.M., Al-Ayed M.S.Z., Qureshi M.A. (2013). Emergence of unusual nonfermenting gram-negative nosocomial pathogens in a Saudi hospital. Jpn. J. Infect. Dis..

[73] Teerawattanapong N., Kengkla K., Dilokthornsakul P., Saokaew S., Apisarnthanarak A., Chaiyakunapruk N. (2017). Prevention and control of multidrug-resistant gram-negative bacteria in adult intensive care units: A systematic review and network meta-analysis. Clin. Infect. Dis..

[74] Garnacho-Montero J., Dimopoulos G., Poulakou G., Akova M., Cisneros J.M., De Waele J., Petrosillo N., Seifert H., Timsit J.F., Vila J. (2015). Task force on management and prevention of *Acinetobacter baumannii* infections in the ICU. Intensive Care Med..

[75] Yamamoto N., Hamaguchi S., Akeda Y., Santanirand P., Kerdsin A., Seki M., Ishii Y., Paveenkittiporn W., Bonomo R.A., Oishi K. (2015). Clinical specimen-direct LAMP: A useful tool for the surveillance of bla oxa-23-positive carbapenem-resistant *Acinetobacter baumannii*. PLoS ONE.

[76] Ulu-Kilic A., Ahmed S., Alp E., Doğanay M. (2013). Challenge of intensive care unit-acquired infections and *Acinetobacter baumannii* in developing countries. OA Crit. Care.

[77] Yusuf I., Adam R.U. (2014). Adopting Chennai declaration strategies in the prevention and control of the spread of multidrug-resistant hospital-acquired bacterial infections in Nigeria: A call to action. J. Glob. Antimicrob. Resist..

[78] Papathanakos G., Andrianopoulos I., Papathanasiou A., Priavali E., Koulenti D., Koulouras V. (2020). Colistin-resistant *Acinetobacter baumannii* bacteremia: A serious threat for critically ill patients. Microorganisms.

[79] Tamma P.D., Aitken S.L., Bonomo R.A., Mathers A.J., van Duin D., Clancy C.J. (2022). Infectious Diseases Society of America Guidance on the Treatment of AmpC β-Lactamase–Producing Enterobacterales, Carbapenem-Resistant *Acinetobacter baumannii*, and *Stenotrophomonas maltophilia* Infections. Clin. Infect. Dis..

[80] Paul M., Carrara E., Retamar P., Tängdén T., Bitterman R., Bonomo R.A., De Waele J., Daikos G.L., Akova M., Harbarth S. (2022). European Society of Clinical Microbiology and Infectious Diseases (ESCMID) guidelines for the treatment of infections caused by multidrug-resistant Gram-negative bacilli (endorsed by European society of intensive care medicine). Clin. Microbiol. Infect..

[81] Abdul-Mutakabbir J.C., Griffith N.C., Shields R.K., Tverdek F.P., Escobar Z.K. (2021). Contemporary perspective on the treatment of *Acinetobacter baumannii* infections: Insights from the Society of Infectious Diseases Pharmacists. Infect. Dis. Ther..

[82] Shields R.K., Paterson D.L., Tamma P.D. (2023). Navigating Available Treatment Options for Carbapenem-Resistant *Acinetobacter baumannii*-calcoaceticus Complex Infections. Clin. Infect. Dis..

[83] Wei J., Peng N., Liang Y., Li K., Li Y. (2020). Phage therapy: Consider the past, embrace the future. Appl. Sci..

[84] Rima M., Rima M., Fajloun Z., Sabatier J.-M., Bechinger B., Naas T. (2021). Antimicrobial peptides: A potent alternative to antibiotics. Antibiotics.

[85] Zhang Y., Lin Y., Galgano S., Houdijk J., Xie W., Jin Y., Lin J., Song W., Fu Y., Li X. (2022). Recent Progress in Phage Therapy to Modulate Multidrug-Resistant *Acinetobacter baumannii*, including in Human and Poultry. Antibiotics.

[86] Yuan Y., Wang L., Li X., Tan D., Cong C., Xu Y. (2019). Efficacy of a phage cocktail in controlling phage resistance development in multidrug resistant *Acinetobacter baumannii*. Virus Res..

[87] Singh R., Capalash N., Sharma P. (2022). Vaccine development to control the rising scourge of antibiotic-resistant *Acinetobacter baumannii*: A systematic review. Biotech.

[88] Mba I.E., Sharndama H.C., Anyaegbunam Z.K.G., Anekpo C.C., Amadi B.C., Morumda D., Doowuese Y., Ihezuo U.J., Chukwukelu J.U., Okeke O.P. (2023). Vaccine development for bacterial pathogens: Advances, challenges and prospects. Trop. Med. Int. Health.

[89] McConnell M.J., Actis L., Pachón J. (2013). *Acinetobacter baumannii*: Human infections, factors contributing to pathogenesis and animal models. FEMS Microbiol. Rev..

[90] Gellings P.S., Wilkins A.A., Morici L.A. (2020). Recent advances in the pursuit of an effective *Acinetobacter baumannii* vaccine. Pathogens.

[91] García-Quintanilla M., Pulido M.R., Pachon J., McConnell M.J. (2014). Immunization with lipopolysaccharide-deficient whole cells provides protective immunity in an experimental mouse model of *Acinetobacter baumannii* infection. PLoS ONE.

[92] KuoLee R., Harris G., Yan H., Xu H.H., Conlan W.J., Patel G.B., Chen W. (2015). Intranasal immunization protects against *Acinetobacter baumannii*-associated pneumonia in mice. Vaccine.

[93] Pulido M.R., García-Quintanilla M., Pachón J., McConnell M.J. (2020). A lipopolysaccharide-free outer membrane vesicle vaccine protects against *Acinetobacter baumannii* infection. Vaccine.

[94] Luo G., Lin L., Ibrahim A.S., Baquir B., Pantapalangkoor P., Bonomo R.A., Doi Y., Adams M.D., Russo T.A., Spellberg B. (2012). Active and passive immunization protects against lethal, extreme drug resistant-*Acinetobacter baumannii* infection. PLoS ONE.

[95] Chen W. (2015). Current advances and challenges in the development of Acinetobacter vaccines. Hum. Vaccines Immunother..

[96] Sheweita S., Batah A., Ghazy A., Hussein A., Amara A. (2019). A new strain of *Acinetobacter baumannii* and characterization of its ghost as a candidate vaccine. J. Infect. Public Health.

[97] Tabrizi C.A., Walcher P., Mayr U.B., Stiedl T., Binder M., McGrath J., Lubitz W. (2004). Bacterial ghosts–biological particles as delivery systems for antigens, nucleic acids and drugs. Curr. Opin. Biotechnol..

[98] Cabral M.P., García P., Beceiro A., Rumbo C., Pérez A., Moscoso M., Bou G. (2017). Design of live attenuated bacterial vaccines based on D-glutamate auxotrophy. Nat. Commun..

[99] Ainsworth S., Ketter P.M., Yu J.-J., Grimm R.C., May H.C., Cap A.P., Chambers J.P., Guentzel M.N., Arulanandam B.P. (2017). Vaccination with a live attenuated *Acinetobacter baumannii* deficient in thioredoxin provides protection against systemic Acinetobacter infection. Vaccine.

[100] Zeller T., Klug G. (2006). Thioredoxins in bacteria: Functions in oxidative stress response and regulation of thioredoxin genes. Naturwissenschaften.

[101] Huang W., Zhang Q., Li W., Chen Y., Shu C., Li Q., Zhou J., Ye C., Bai H., Sun W. (2019). Anti-outer membrane vesicle antibodies increase antibiotic sensitivity of pan-drug-resistant *Acinetobacter baumannii*. Front. Microbiol..

[102] Jin J.S., Kwon S.-O., Moon D.C., Gurung M., Lee J.H., Kim S.I., Lee J.C. (2011). *Acinetobacter baumannii* secretes cytotoxic outer membrane protein A via outer membrane vesicles. PLoS ONE.

[103] Ellis T.N., Kuehn M.J. (2010). Virulence and immunomodulatory roles of bacterial outer membrane vesicles. Microbiol. Mol. Biol. Rev..

[104] Alaniz R.C., Deatherage B.L., Lara J.C., Cookson B.T. (2007). Membrane vesicles are immunogenic facsimiles of *Salmonella typhimurium* that potently activate dendritic cells, prime B and T cell responses, and stimulate protective immunity in vivo. J. Immunol..

[105] McBroom A.J., Kuehn M.J. (2007). Release of outer membrane vesicles by Gram-negative bacteria is a novel envelope stress response. Mol. Microbiol..

[106] Deatherage B.L., Lara J.C., Bergsbaken T., Barrett S.L.R., Lara S., Cookson B.T. (2009). Biogenesis of bacterial membrane vesicles. Mol. Microbiol..

[107] Kim O.Y., Hong B.S., Park K.-S., Yoon Y.J., Choi S.J., Lee W.H., Roh T.-Y., Lötvall J., Kim Y.-K., Gho Y.S. (2013). Immunization with *Escherichia coli* outer membrane vesicles protects bacteria-induced lethality via Th1 and Th17 cell responses. J. Immunol..

[108] Wang H., Liang K., Kong Q., Liu Q. (2019). Immunization with outer membrane vesicles of avian pathogenic Escherichia coli O78 induces protective immunity in chickens. Vet. Microbiol..

[109] Gasperini G., Biagini M., Arato V., Gianfaldoni C., Vadi A., Norais N., Bensi G., Delany I., Pizza M., Arico B. (2018). Outer membrane vesicles (OMV)-based and proteomics-driven antigen selection identifies novel factors contributing to Bordetella pertussis adhesion to epithelial cells. Mol. Cell. Proteom..

[110] Nieves W., Petersen H., Judy B.M., Blumentritt C.A., Russell-Lodrigue K., Roy C.J., Torres A.G., Morici L.A. (2014). A *Burkholderia upseudomallei* outer membrane vesicle vaccine provides protection against lethal sepsis. Clin. Vaccine Immunol..

[111] Baker S.M., Davitt C.J., Motyka N., Kikendall N.L., Russell-Lodrigue K., Roy C.J., Morici L.A. (2017). A *Burkholderia pseudomallei* outer membrane vesicle vaccine provides cross protection against inhalational glanders in mice and non-human primates. Vaccines.

[112] Pulido M.R., García-Quintanilla M., Pachón J., McConnell M.J. (2018). Immunization with lipopolysaccharide-free outer membrane complexes protects against *Acinetobacter baumannii* infection. Vaccine.

[113] Ma C., McClean S. (2021). Mapping global prevalence of *Acinetobacter baumannii* and recent vaccine development to tackle it. Vaccines.

[114] Yang A.Q., Yang H.Y., Guo S.J., Xie Y.E. (2019). MF59 adjuvant enhances the immunogenicity and protective immunity of the *OmpK*/*Omp22* fusion protein from *Acineterbacter baumannii* through intratracheal inoculation in mice. Scand. J. Immunol..

[115] García-Patiño M.G., García-Contreras R., Licona-Limón P. (2017). The immune response against *Acinetobacter baumannii*, an emerging pathogen in nosocomial infections. Front. Immunol..

[116] Lei L., Yang F., Zou J., Jing H., Zhang J., Xu W., Zou Q., Zhang J., Wang X. (2019). DNA vaccine encoding OmpA and Pal from *Acinetobacter baumannii* efficiently protects mice against pulmonary infection. Mol. Biol. Rep..

[117] Lau Y.T., Tan H.S. (2023). *Acinetobacter baumannii* subunit vaccines: Recent progress and challenges. Crit. Rev. Microbiol..

[118] Nie D., Hu Y., Chen Z., Li M., Hou Z., Luo X., Mao X., Xue X. (2020). Outer membrane protein A (*OmpA*) as a potential therapeutic target for *Acinetobacter baumannii* infection. J. Biomed. Sci..

[119] Hounsome J.D., Baillie S., Noofeli M., Riboldi-Tunnicliffe A., Burchmore R.J., Isaacs N.W., Davies R.L. (2011). Outer membrane protein A of bovine and ovine isolates of *Mannheimia haemolytica* is surface exposed and contains host species-specific epitopes. Infect. Immun..

[120] Smith M.G., Gianoulis T.A., Pukatzki S., Mekalanos J.J., Ornston L.N., Gerstein M., Snyder M. (2007). New insights into *Acinetobacter baumannii* pathogenesis revealed by high-density pyrosequencing and transposon mutagenesis. Genes Dev..

